# Real-Time Determination of Photosynthesis, Transpiration, Water-Use Efficiency and Gene Expression of Two *Sorghum bicolor* (Moench) Genotypes Subjected to Dry-Down

**DOI:** 10.3389/fpls.2017.00932

**Published:** 2017-05-31

**Authors:** Alessandra Fracasso, Eugenio Magnanini, Adriano Marocco, Stefano Amaducci

**Affiliations:** Department of Sustainable Crop Production, Università Cattolica del Sacro CuorePiacenza, Italy

**Keywords:** drought stress tolerance, whole canopy water use efficiency, gene expression dynamics, gas exchange, *Sorghum bicolor*

## Abstract

Plant growth and productivity are strongly affected by limited water availability in drought prone environments. The current climate change scenario, characterized by long periods without precipitations followed by short but intense rainfall, forces plants to implement different strategies to cope with drought stress. Understanding how plants use water during periods of limited water availability is of primary importance to identify and select the best adapted genotypes to a certain environment. Two sorghum genotypes IS22330 and IS20351, previously characterized as drought tolerant and drought sensitive genotypes, were subjected to progressive drought stress through a dry-down experiment. A whole-canopy multi-chamber system was used to determine the *in vivo* water use efficiency (WUE). This system records whole-canopy net photosynthetic and transpiration rate of 12 chambers five times per hour allowing the calculation of whole-canopy instantaneous WUE daily trends. Daily net photosynthesis and transpiration rates were coupled with gene expression dynamics of five drought related genes. Under drought stress, the tolerant genotype increased expression level for all the genes analyzed, whilst the opposite trend was highlighted by the drought sensitive genotype. Correlation between gene expression dynamics and gas exchange measurements allowed to identify three genes as valuable candidate to assess drought tolerance in sorghum.

## Introduction

Drought and problems related to water supply will be exacerbated under the current climate change scenario (ICPP, 2014). Efforts to increase water productivity in agriculture are essential to support an increasing demand for food, feed and energy. Breeding crops for high water use efficiency (WUE) could be a solution to secure adequate water for agriculture and to address food security (Hall and Richards, 2013), pursuing the slogan “more crop per drop” (Monaghan et al., 2013).

WUE does not have a single precise definition (Bacon, 2009) and its scale and unit of measurement vary greatly following the point of view of physiologists and agronomists (Condon et al., 2002). At single leaf level, WUE can be defined as the ratio between net assimilation and transpiration (instantaneous WUE) or as the ratio between net assimilation and stomatal conductance (intrinsic WUE). At canopy or field level, it is calculated as the yield of harvested product achieved from the water available to the crop through precipitation and/or irrigation (Condon et al., 2002). Lack of significant correlation between WUE measured at leaf level and WUE measured at whole canopy level has been reported (Tomás et al., 2012) and it was attributed to the inadequacy of single-leaf measurements to represent the spatial variability occurred at the whole-canopy level, or to the inadequacy of single-leaf measurements to take into account nocturnal water loss and respiration (Escalona et al., 2012), and changes in dry matter partitioning among different sinks (e.i. shoot or root) (Tomás et al., 2014). In addition, punctual single-leaf measurements are inadequate to represent the temporal variability that occurs along the day and during the entire crop growing period. For these reasons, we choose to investigate the instantaneous WUE at canopy level using an automated multi-chamber whole-canopy system that allows for continuous monitoring of net photosynthetic (Pn) and transpiration (E) rate. Numerous researches have been carried out on trees using whole-canopy gas exchange systems (Dragoni et al., 2005; Intrigliolo et al., 2009; Rodrigues et al., 2016), but very few are reported on field crops (Timlin et al., 2006; Cantore et al., 2009; Cabrera-Bosquet et al., 2011) and none on sorghum.

*Sorghum bicolor* (Moench) is a C4 multipurpose crop valuable for food, feed, fiber and fuel production (Paterson et al., 2005), and well adapted to drought-prone environments due to its deep and dense root system (Stone et al., 2001), its thick leaf wax (Cannon and Kummerow, 1957), its osmotic adjustment (Dugas et al., 2011) and its high photosynthetic efficiency under drought (Zegada-Lizarazu et al., 2012). For this sorghum was also recently considered as a sustainable crop for biogas production in marginal environments (Amaducci et al., 2016).

Breeding for improved WUE crops was pursued also at molecular level. For example, wheat plant transformed with *HVA1* gene from barley showed modified WUE (Sivamani et al., 2000); the same approach was used to transform rice plants with the salt and drought tolerant gene *HARDY* from Arabidopsis (Karaba et al., 2007) in order to improve WUE. In addition, in Arabidopsis was also identified on chromosome 2 a QTL for WUE and assigned to the ERECTA gene (Masle et al., 2005).

In the present study CO_2_ and water vapor gas exchange of two sorghum genotypes IS20351 and IS22330, previously characterized as drought sensitive and drought tolerant respectively (Fracasso et al., 2016b), were continuously monitored for 2 weeks in 2015. Whole-canopy instantaneous WUE (wcWUE_i_) was calculated as the ratio between whole-canopy Pn and E from the onset of water stress until the end of the experiment (11th leaf). The expression trend of five drought related genes was monitored every 4 h on plants that had been subjected to severe drought stress for 7 days. The objective of the present study was: (a) assess the diurnal gas exchange dynamics of two sorghum genotypes, explore the variation in Pn, E, and wcWUE_i_ under increasing drought stress and confirm the strategies adopted by the sorghum genotypes to cope with drought stress; (b) establish which gene could better explain the variation in Pn, E, and wcWUE_i_ encountered during the day and which gene could be used as a proxy for drought tolerance and a potential candidate for genetic improvement in sorghum.

## Materials and methods

### Plant material, growth conditions, and experimental design

Two sorghum genotypes, belonging to the *durra* race and characterized as drought sensitive and drought tolerant in a previous study (Fracasso et al., 2016b), were cultivated in 2015 in a dry-down pot experiment. The genotypes, IS20351 and IS22330, are part of the germoplasm collection of CIRAD and were provided by the CRB-T (Centre de Resources Biologiques Tropicales) CIRAD Montpellier. Germination of seeds was carried out in Petri dishes at 25°C and in dark conditions for 3 days. Germinated seeds were planted in plastic pots (0.016 m^3^ capacity), filled with a base layer of sand to guarantee drainage and 8 kg of a soil mixture (topsoil and zeolite 3:1 in volume). Each pot was planted with five germinated seeds, that were thinned to one healthy plant per pot when the 4th leaf appeared.

The plants were grown in a growth chamber with 13 h of light and 27–23°C day-night temperature until the 7th leaf appeared on the main stem. At this stage the leaf area (LA) of each plant was calculated every week following the procedure described in Fracasso et al. (2016a). To perform whole-canopy WUE_i_ determination, the plants were then moved in a greenhouse under LED lamps. Light intensity under each LED lamp was measured with a ceptometer (AccuPAR LP80, Decagon Devices, USA) and decreased from 1,500 micromoles PPFD at 20 cm from the LED lamp, to 700 micromoles at 60 cm from the LED lamp with a light extinction coefficient, calculated according the Lambert and Beer Law, of 7.33.

Six plants for each genotype were randomly assigned to a well-watered (WW) or to a water-stressed (WS) treatment. All the plants were kept above 0.7 FTSW values until the 39th DAE (day after emergence) by supplying manually a daily amount of water calculated following the gravimetric method. Starting on the 40th DAE a progressive water deficit was imposed to the WS plants withholding irrigation. Water deficit was represented as the Fraction of Transpirable Soil Water (FTSW) calculated as in Luquet et al. (2008):

$$\begin{array}{l} FTSW=\frac{ASWC}{TTSW}=\;\frac{SWC-WP}{FC-WP} \end{array}$$

Where Total Transpirable Soil Water (TTSW) content was determined as the difference between the Soil Water Content (SWC) at Field Capacity (FC) and that at Wilting Point (WP). Both FC and WP were determined in a preliminary experiment (data not shown). FC was the soil water content left in the soil after complete drainage of excess water while WP was the residual water left in the soil after sorghum plants were grown until complete wilting. ASWC was the Available Soil Water Content, calculated subtracting the soil water content at WP from the actual soil water content SWC. During the experiment six pots were weighed by an automated weigh system.

On the 38th DAE, the top of the pots was covered with PVC bags. A little slit was cut at the bottom of the plastic bag to allow the sorghum plant to grow through it. The slit was sealed with adhesive packing tape around plant collars to avoid evaporation losses from the soil surface. The plants were put inside the multi-chamber gas-exchange apparatus for gas exchange measurements on 44th DAE. The dry-down phase was followed by 7 days in which FTSW in the soil was keep stable at 0.15, providing the daily transpired water to WS plants at the end of each day. On 60th DAE, after seven days at 0.15 FTSW, leaf samples from three biological replicates were collected at 4 h intervals from 6 a.m. to 6 p.m. 2 g of leaf tissue from the last two fully opened leaves were immediately frozen in liquid nitrogen for qRT-PCR analysis.

### The multi-chamber gas exchange system

Whole-canopy photosynthesis and transpiration were determined with a self-assembled multi-chamber gas exchange apparatus. It consists of 12 cylindrical chambers made of flexible plastic polyethylene with polymethylmethacrylate tops (allowing 93 and 87% of light transmission respectively and no alteration of light spectrum). The volume of the chambers is variable and during the present experiment was 0.157 m^3^ ± 0.06 (0.8 m height, 0.5 m diameter). A CIRAS-DC double channel absolute CO_2_/H_2_O infrared gas analyser (PP-System, Amesbury, MA) combined to a CR1000 datalogger wired to an AM16/32B Multiplexer (Campbell Scientific, Logan, UT) are used to analyse air at the inlet and outlet of each chamber following a program described in Poni et al. (2014). Air is drawn from outside the greenhouse at 3 m above ground, it is forced, with two centrifugal blowers (Vorticent C25/2M Vortice, Milan, Italy), through a buffer tank (0.44 m^3^ capacity) to ensure the stability of inlet CO_2_ concentration and then it is blown to the chambers through 50 mm diameter rigid plastic pipes. Air sampling is switched from one chamber to another at programmed time intervals using a set of solenoid valves (model 177 B04/Z610, Sirai, Bussero, Italy). The air flow rate of each chamber is controlled by a baffle and measured at least 50 cm downstream the baffle itself with a digital manometer (Testo 510, Farnell, Lainate, Italy) according to a flow-restriction method (Osborne, 1977). A 22 mm inner-diameter ring was inserted inside the 50 mm pipe in order to achieve the flow restriction; the pressure drop of 9 mm water column, measured upstream and downstream from the ring, corresponded to 0.0028 m^3^s^−1^.

Two additional rotary vane pumps (model G 6/01-K-LCL; Gardner, Denver Thomas, Pucheim, Germany) with 33.3 cm^3^s^−1^ of flow rate were added before CIRAS-DC to speed the air flushing inside the sampling tubes and ensure the complete air exchange inside the CIRAS-DC when AM16/32B Multiplexer switches from one chamber to another one.

The ambient (inlet) air temperature and the air temperature of each chamber (outlet) were measured by shielded external/internal (1/0.2 mm) diameter PFA-Teflon insulated type-T thermocouples (TC). The system was connected to an external laptop computer for checking and downloading data from the datalogger support software package LoggerNet (Campbell Scientific, Logan, UT).

### The whole-canopy gas exchange measurements

Whole-canopy net photosynthetic rate (Pn) and transpiration rate (E) values were measured continuously, 24 h per day, using the multi-chamber system for the entire duration of the experiment, from the 44th DAE to the 59th DAE. The flow rate fed to the chambers was set at 0.0028 m^3^s^−1^ and kept constant throughout the entire duration of the experiment. The polyethylene chambers had a volume of 0.157 m^3^ ± 0.06, so the complete volume air change occurred at an interval of ~60 s. Pn (μmol s^−1^ m^−2^) and E (mmol s^−1^ m^−2^) were calculated from flow rates and CO_2_ and water vapor differentials using the formula provided in Long and Hällgren (1985).

### RNA extraction and real-time reverse transcription-PCR analysis

Leaf tissues were ground to a fine powder with liquid nitrogen using sterile mortars and pestles. RNA extraction was performed on 100 mg of plant material using TRIZOL reagent (EuroGold Trifast, EuroClone Ltd., Torquay, UK), purification was performed using RNA CleanUp protocol (Qiagen, Hilden, Germany) and treating the samples with RNase-Free DNase (Invitrogen, Carlsbad, CA) to avoid genomic DNA contamination during RNA purification. The concentration of total RNA fraction in each sample was estimated by spectrophotometric assay using Qubit 1.0 fluorometer (Invitrogen, Carlsbad, CA, USA). The cDNA was synthesized from total RNA following the iScript cDNA Synthesis Kit (Bio-Rad, Richmond, CA) and used for quantitative real-time PCR. Real-time PCR (CFX96TM Real-Time PCR machine; Bio-Rad Lab-oratories, Inc., Hercules, USA) was performed on the samples, and relative gene expression was determined using the 2^−ΔΔCt^ Ct method (Livak and Schmittgen, 2001). The amplification reaction was optimized in a final volume of 20 μl containing 20 ng cDNA, 10 μl of 2X iQ SYBR Green Supermix (Bio-Rad, Richmond, CA) and 4 μM of each primer. The amplification was carried out according to the following protocol: 95°C for 3 min (1 cycle), 95°C for 15 s, 60°C for 1 min (39 cycle), melting curve analysis, with an increase in temperature of 0.5°C per s from 65 to 95°C. Three technical replicates were carried out for each of the three biological replicates tested at each stress condition for all the genotypes. Expression analysis was performed on five genes identified as potential targets of stomatal control and as responsible of photosynthesis (Pasini et al., 2014). Tubulin gene was used as reference gene to normalize relative quantification (Table 1). Expression ratio sand fold change (FC) were calculated using the 2^−ΔΔCt^ method.

**Table 1:** Gene name, annotation, function, primer sequences, and product size, QTL mapping and references reported in literature of genes used in quantitative real-time PCR.

**Gene name**	**Gene annotation**	**Gene function**	**Primer (5′–3′)**		**Product size**	**QTL mapping**	**Literature**
Sobic.003G292400.1	SbNADP-ME	C4 carbon fixation	For	AGAAACTGGCCAGAGAAGAGTA	173	QSTG13_3	Laporte et al., 2002; Guo et al., 2009
			Rev	ACATCAATGGTAATAGGCAGGC			Doubnerová and Ryšlavá, 2011
Sobic.003G234600.1	SbCA	C4 carbon fixation	For	CGTAATATTGCCAGCCTAGTCC	182	–	Hu et al., 2010; Studer et al., 2014
			Rev	AAGTGGTAGGTGTGTGGTCTAT			
Sobic.001G298100.1	SbDHN	Osmolyte biosynthesis	For	TGTGAAGGCAGGTGAAACAG	105	QSTG2_9; QSTG3_9	Brini et al., 2007; Xiao et al., 2007
			Rev	AGGCTGGCGTAGTACACATC			Gosal et al., 2009.
Sobic.001G184100.1	SbK*^+^*O	Osmotic stress potassium transporter	For	TTTTGCTGATCTCGGGTACTTC	167	–	
			Rev	AAGAGCTTGGAACCGAATTGAA			
Sobic.010G020600.1	SbERECTA	Receptor-like protein kinase 5 precursor	For	GATACTTGGCAACCTGTCCTAC	125	–	
			Rev	GTTCATTGTCATTCAGCTGCAG			
Sobic.006G181800.2	Tubulin C-terminal	–	For	AAATAGCTGACTGGGCAGATTC	118	–	
			Rev	TTCTGCAAAGCCAAGTCCATAA			

## Statistical analysis

One-way ANOVA was carried out and, when the F-test was significant, mean separation was performed by the *t*-test at *P* < 0.05 and *P* < 0.01. Degree of variation around means is given as standard error (SE).

## Results

### Characterization of drought dynamics and whole-canopy gas exchange

**Figure 1:**
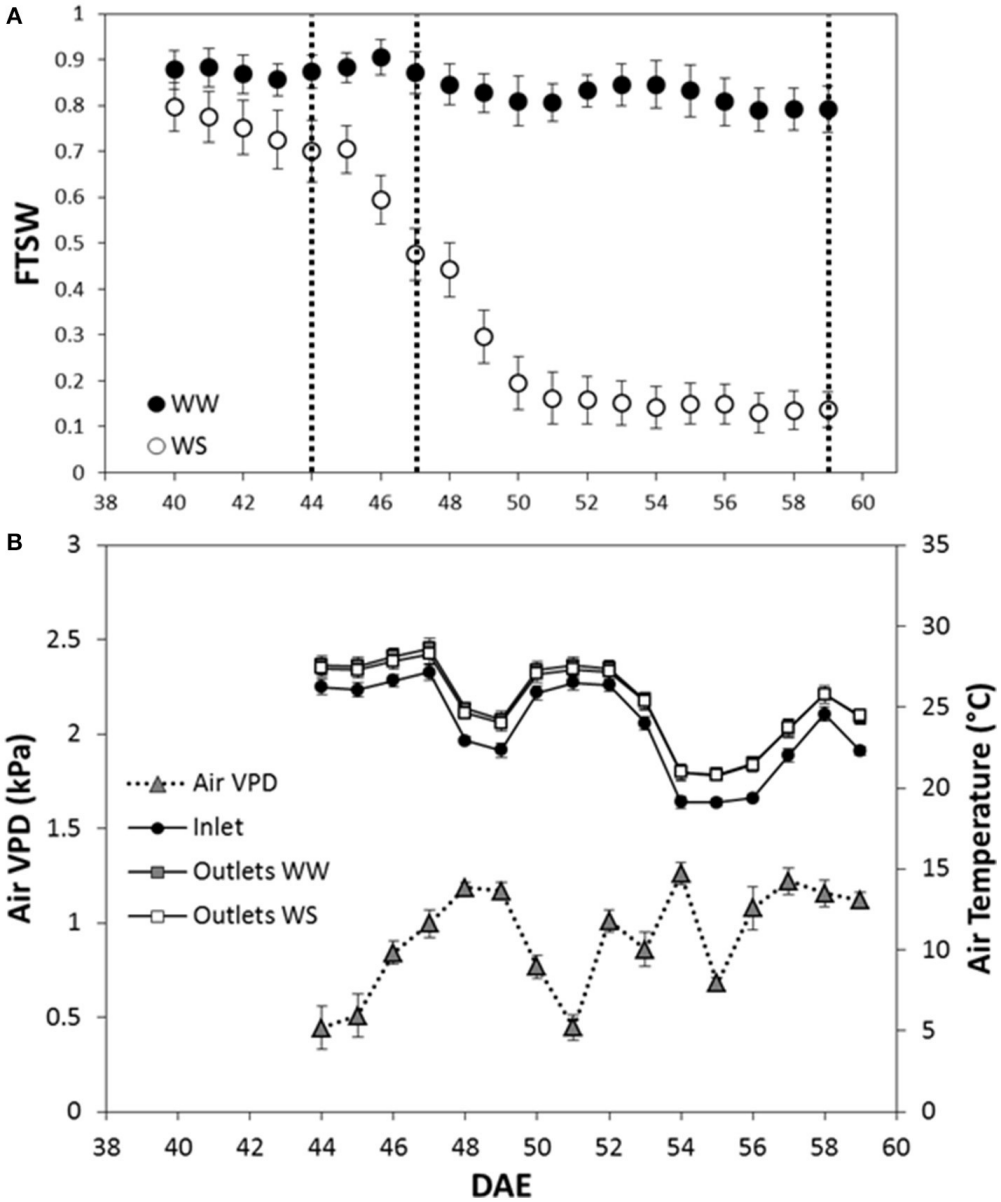
Fraction of transpirable soil water (FTSW) dynamics, air temperature and air VPD recorded during the dry-down experiment carried out on two *Sorghum bicolor* genotypes. **(A)** The FTSW dynamics is presented in function of the day after plant emergence (DAE); the empty circles represent the plants under water stress (WS) while the full circles the well-watered (WW) plants. Vertical dotted lines indicate the date in which hourly Pn, E and wcWUE_i_ were analyzed. Mean of 3 ± SE. **(B)** Air temperature of inlet, and outlet of WW and WS plants, and air VPD. Mean 3 ± SE.

FTSW decreased for the water stressed (WS) plants after irrigation was with-held (40th DAE) and, when 0.15 FTSW was reached (52nd DAE), FTSW was kept constant to 0.15 for 7 days (Figure 1A).

The multi-chamber system ran continuously for 2 weeks from the 44th DAE to the 59th DAE.

The system recorded the air temperature entering (inlet) and leaving (outlet) the chambers. Mean daily temperature of inlet air was 23.7°C; mean daily temperature of outlet air was 25.4°C and 25.3°C at the outlet of chambers hosting WW and WS plants respectively (Figure 1B). Air temperature was used to calculate air VPD from the 44th DAE to the 59th DAE as reported in Figure 1B.

**Figure 2:**
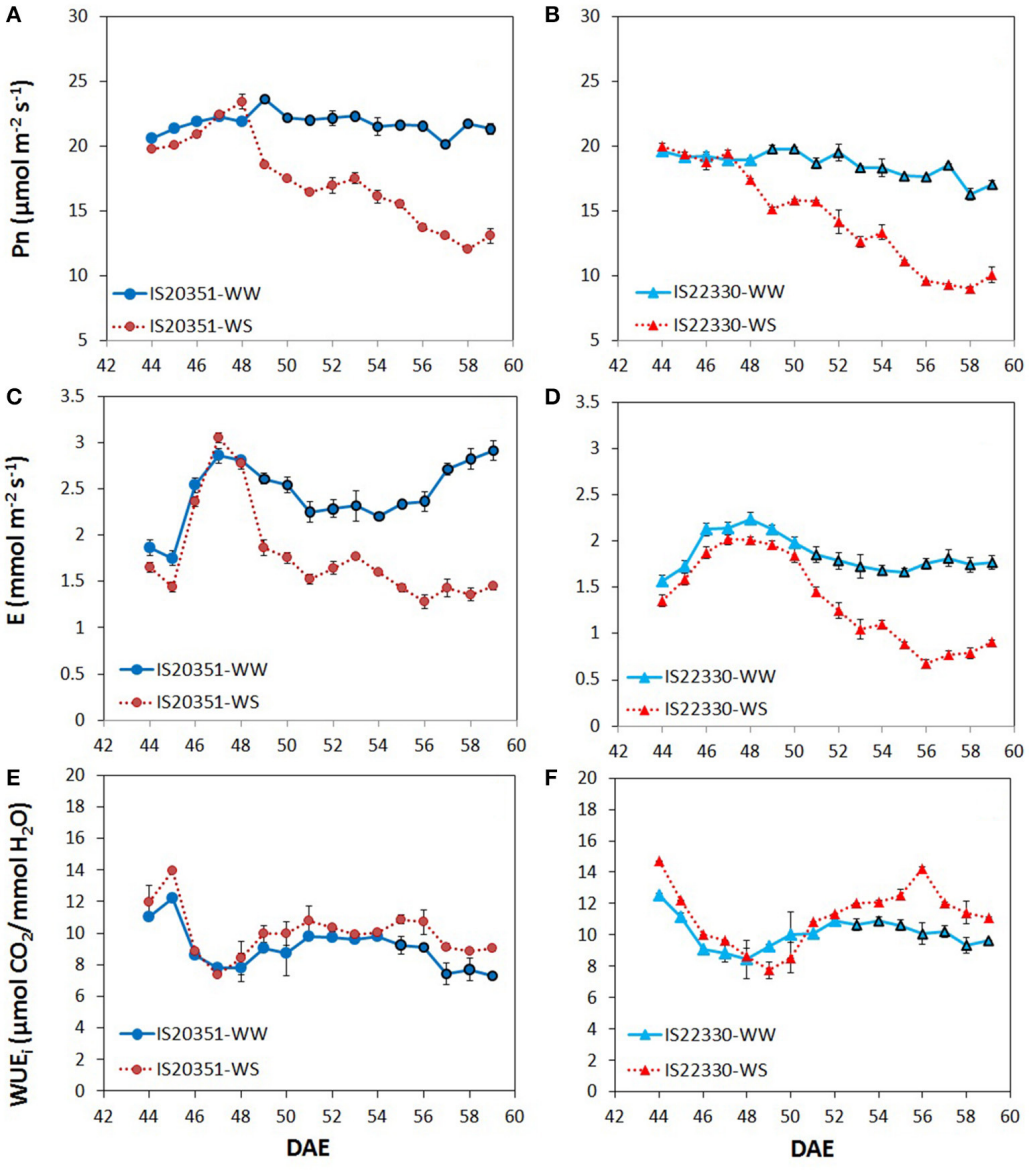
Net photosynthetic rate, transpiration rate and whole-canopy WUE_i_ recorded during the experiment for the two genotype IS20351 and IS22330 under well-watered (WW) and water-stressed (WS) conditions. **(A,B)** net photosynthetic rate (Pn, μmol m^−2^ s^−1^) recorded from 44th DAE to 59th DAE for IS20351 and IS22330 respectively; **(C,D)** transpiration rate (E, mmol m^−2^ s^−1^) recorded from 44th DAE to 59th DAE for IS20351 and IS22330 respectively; **(E,F)** whole-canopy WUE_i_ (wcWUE_i_, μmol CO_2_/ mmol H_2_O) recorded from 44th DAE to 59th DAE for IS20351 and IS22330 respectively. The red symbols represent the plants under water stress (WS) while the blue circles the well-watered (WW) plants. Symbols with highlighted black outline are statistically significant (*p* < 0.05). Mean of 3 ± SE.

Mean daily Pn for WW IS20351 and WW IS22330 plants was constant for the entire duration of the experiment (Figures 2A,B). In both genotypes, Pn under WS conditions decreased proportionally to the decrease of FTSW, starting from the 49th DAE (Figures 2A,B). On average, Pn in the drought tolerant genotype IS22330 was significantly lower than Pn in the sensitive genotype IS20351 under both WW and WS conditions (*p* < 0.05). A similar trend was also observed for E (Figures 2C,D). Under WW conditions, the drought tolerant genotype IS22330 had lower E compared to the drought sensitive IS20351 (1.85 and 2.44 mmol m^2^ s^−1^ for IS22330 and IS20351 respectively, *p* < 0.01). Under WS conditions, from the 49th DAE to the 59th DAE, E decreased linearly for both genotypes, by about 22% (1.44 mmol m^2^ s^−1^) and 27% (1.77 mmol m^2^ s^−1^) for IS22330 and IS20351 respectively.

Whole-canopy instantaneous WUE (wcWUE_i_) expressed as Pn/E ratio (μmol CO_2_/ mmol H_2_O) was similar for both genotypes and water treatments at the beginning of the experiment and it became statistically different between water treatments (*p* < 0.05) only under limiting FTSW (0.15 FTSW). In IS20351, wcWUE_i_ was higher in WS plants than in WW plants from the 55th DAE onwards (Figure 2E, *p* < 0.05), while in IS22330 wcWUE_i_ started to be significantly different between WS and WW plants from the 53rd DAE (Figure 2F, *p* < 0.01). On average wcWUE_i_ under WS conditions was 10.0 and 11.1 μmol CO_2_/mmol H_2_O for IS20351 and IS22330 respectively.

**Figure 3:**
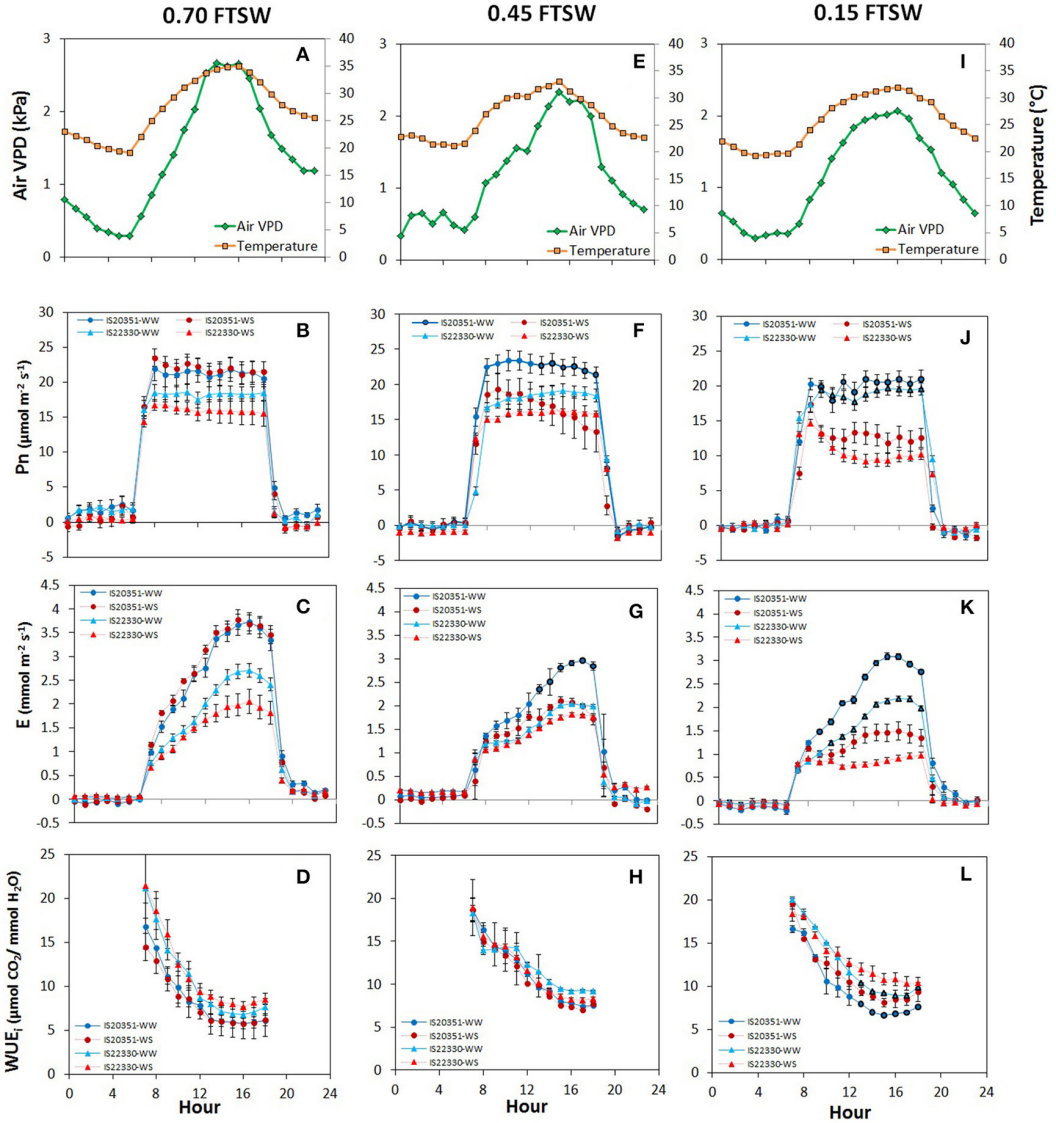
Daily air VPD and air temperature **(A,E,I)**, net photosynthetic rate **(B,F,J)**, transpiration rate **(C,G,K)**, and whole-canopy WUE_i_ **(D,H,L)** recorded at 0.70 (44th DAE), 0.45 (47th DAE) and 0.15 FTSW (59th DAE) for the two genotype IS20351 (circles) and IS22330 (triangles) under well-watered (WW) and water-stressed (WS) conditions. The red symbols represent the plants under water stress (WS) while the blue circles the well-watered (WW) plants. Symbols with highlighted black outline are statistically significant (*p* < 0.05).

Diurnal variation of air VPD, temperature, Pn, E and wcWUE_i_ reported in Figure 3 is representative of three moments of the dry-down experiment: before the water stress occurred (0.70 FTSW for WS plants, 44th DAE, Figures 3A–D), at 0.45 FTSW (47th DAE, Figures 3E–H) and at 0.15 FTSW (59th DAE, Figures 3I,L–N). Diurnal direct and diffuse light was constant for the entire duration of the experiment, from 7 a.m. to 7 p.m., when LED lights were on.

Before drought stress occurred (0.70 FTSW, 44th DAE) Pn was constant throughout the day and had similar values in both genotypes and water treatments (Figure 3B). Daily E increased during the day reaching its maximum at 3 p.m. (Figure 3C) when the maximum air VPD occurred (Figure 3A). wcWUE_i_ decreased progressively throughout the day without significant differences between treatments and genotypes (Figure 3D).

On the 47th DAE (0.45 FTSW) starting from 1 pm, the WS plants of the drought sensitive genotype IS20351 reduced Pn by 26% compared to the WW IS20351 plants (Figure 3F, *p* < 0.01). Pn of WS IS22330 plants was not reduced compared to WW IS22330 plants (Figure 3F) and it was stable during the day. The daily trend of E was the same recorded on the 44th DAE with differences in the absolute values due to the lower FTSW (Figure 3G). At 0.45 FTSW, E of WS IS20351 plants decreased on average by 24% compared to WW IS20351 plants; the decrease in E of WS IS20351 plants started from 12 p.m. and lasted until 6 p.m. (*p* < 0.01). At the same level of FTSW, E of WS IS22330 plants did not decrease compared to WW IS22330 plants; the E daily trend was constant throughout the day from 7 a.m. to 7 p.m. (Figure 3G). wcWUE_i_ at 0.45 FTSW was 24% higher than WUE_i_ recorded at 0.70 FTSW on the 44th DAE (*p* < 0.1), but no differences between treatments or genotypes were identified (Figure 3H).

After 7 days at 0.15 FTSW (59th DAE), Pn of WS plants was on average reduced by 35% in IS20351 and by 40% in IS22330 compared to the WW plants (*p* < 0.01). The Pn reduction started for both the genotypes from 9 a.m. (Figure 3L) when VPD started to increase (Figure 3I, *p* < 0.01). E was largely affected at low FTSW and it decreased: by 45 and 47% with respect to WW conditions respectively for IS20351 and IS22330 (Figure 3M). Daily course of E was different for WS plants of IS20351 and IS22330: in the drought sensitive genotype IS20351 E increased from 12 p.m. to 6 p.m. (Figure 3M, *p* < 0.01) following the VPD trend, while in the drought tolerant genotype IS22330 E was stable throughout the day (Figure 3M). As a consequence, at 0.15 FTSW (59th DAE), the difference in wcWUE_i_ between WS and WW plants was statistically significant (Figure 3N, *p* < 0.05) for both genotypes. Furthermore, the difference in wcWUE_i_ of WS plants was also significant between the two genotypes being wcWUE_i_ 11.28 and 13.21 μmol CO_2_/mmol H_2_O respectively for IS20351 and IS22330 (*p* < 0.05).

### Gene expression dynamics

**Figure 4:**
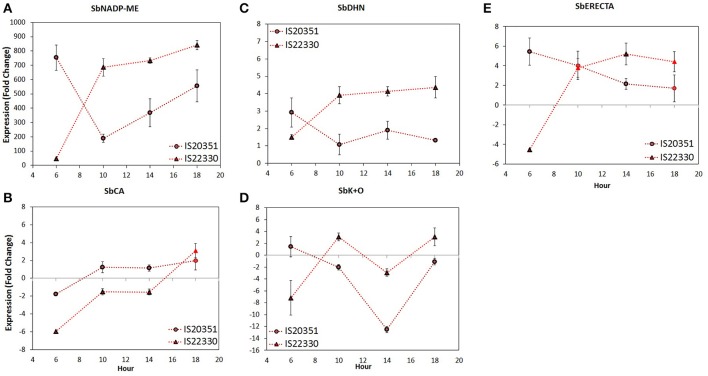
Gene expression level of *SbNADP-ME* **(A)**, *SbCA* **(B)**, *SbDHN* **(C)**, *SbK*+*O* **(D)**, and *SbERECTA* **(E)** recorded on 60th DAE at 0.15 FTSW for the two genotype IS20351 (circles) and IS22330 (triangles) under water-stressed (WS) conditions. Symbols with highlighted black outline are statistically significant (*p* < 0.05). Mean of 9 ± SE for gene expression data.

Previous transcriptomic analysis of sorghum under drought stress and control conditions (Pasini et al., 2014; Fracasso et al., 2016a,b) identified five candidate genes differentially expressed under stress. Their names, annotations, functions and information about co-localization with QTL for drought tolerance are described in Table 1. Expression of these five candidate genes under WS and WW conditions was analyzed using Q-PCR. The gene expression analysis, carried out following the 2^−ΔΔCt^ method, revealed during the day two different gene expression profiles for IS20351 and IS22330 (Figure 4). These profiles could be grouped in two main clusters: (i) in the first one, gene expression profiles increased during the day (it was observed mainly for the drought tolerant genotype IS22330); (ii) in the second one, gene expression profiles decreased during the day (represented mainly by the drought sensitive genotype IS20351). The only exception to this trend was represented by *SbCA* that increased its expression during the day in both genotypes.

The *SbNADP-ME* expression profile of IS22330 showed an abrupt increase from 6 a.m. to 10 a.m., followed by a slight increase until 6 p.m. (*p* < 0.01). For IS20351 an abrupt decrease between 6 a.m. and 10 a.m. was followed by a linear increase from 10 a.m. to 6 p.m. (Figure 4A, *p* < 0.05).

The expression profile of *SbCA*, that were similar for both genotypes, increased slightly from 6 a.m. to 10 a.m. and from 2 p.m. to 6 p.m. (*p* < 0.05), while it was stable between 10 a.m. and 2 p.m. (Figure 4B, *p* < 0.05).

The *SbDHN* expression profile increased for the drought tolerant IS22330 from 6 a.m. to 10 a.m. (*p* < 0.05) and then remained stable for all the day. In the drought sensitive genotype IS20351, *SbDHN* expression profile decreased from 6 a.m. to 10 a.m. (*p* < 0.05) and then it remained stable for all the day. The expression values of IS20351 were 2-times lower than values for IS22330 (Figure 4C).

The expression profile of *SbK*+*O* increased abruptly from 6 a.m. to 10 a.m. in the drought tolerant genotype IS22330 (*p* < 0.01), it decreased at 2 p.m. and increased again at 6 p.m. (Figure 4D, *p* < 0.05). In the drought sensitive genotype IS20351 a slightly decrease was observed from 6 a.m. to 10 a.m. followed by an abrupt decrease at 2 p.m. and an increase at 6 p.m. (Figure 4D, *p* < 0.05).

The *SbERECTA* expression profile abruptly increased from 6 a.m. to 10 a.m. in IS22330 and then it remained stable until 6 p.m. (Figure 4E, *p* < 0.01). On the other hand, in IS20351 a slightly decrease of SbERECTA expression was observed along the day from 6 a.m. to 6 p.m. (n.s.).

On average the expression of *SbNADP-ME, SbDHN, SbK*+*O*, and *SbERECTA* in the drought tolerant IS22330 was higher than in the sensitive genotype IS20351 (*p* < 0.05). The only exception was represented by *SbCA* for which gene expression in the drought sensitive genotype IS20351 was higher than in the drought tolerant IS22330. A Pearson correlation matrix between gene expression and physiological data of Pn, E and wcWUE_i_ recorded on 59th DAE was calculated separately for the two genotypes (Table 2). Positive and strong correlations between physiological data and gene expression were found in the drought tolerant genotype IS22330, whilst negative correlations were found for the drought sensitive IS20351. In particular *SbNADP-ME, SbDHN*, and *SbERECTA* were highly positively correlated with Pn and E in IS22330 whilst they were negatively correlated in IS20351 (Table 2). In IS22330 *SbDHN* and *SbERECTA* positively correlated with *SbNADP-ME*. The same correlation was weaker in the sensitive IS20351 (Table 2). The correlation between *SbCA* and *SbNADP-ME, SbDHN*, and *SbERECTA* was positive in the tolerant IS22330 and negative in the sensitive IS20351 (Table 2). The same was observed for the correlation between *SbK*+*O* and *SbCA* and between *SbK*+*O* and Pn or E (Table 2).

**Table 2:** Values of Pearson correlation performed between physiological measurements and gene expression levels recorded every 4 h for the drought sensitive IS20351 and the drought tolerant IS22330 sorghum genotype.

**IS20351**	**Pn**	**E**	**wcWUE_i_**	**VPD**	***SbNADP-ME***	***SbCA***	***SbDHN***	***SbK+O***	***SbERECTA***
Pn	–								
E	0.96	–							
WUE	−0.56	−0.99	–						
VPD	0.95	0.99	−0.91	–					
*SbNADP-ME*	−0.79	−0.63	−0.79	−0.64	–				
*SbCA*	0.97	0.95	−0.32	0.91	−0.65	–			
*SbDHN*	−0.90	−0.77	−0.81	−0.71	0.79	−0.91	–		
*SbK+O*	−0.56	−0.67	0.54	−0.75	0.47	−0.41	0.16	–	
*SbERECTA*	−0.82	−0.93	0.96	−0.91	0.30	−0.87	0.59	0.57	–
**IS22330**	**Pn**	**E**	**wcWUE**_i_	**VPD**	***SbNADP-ME***	***SbCA***	***SbDHN***	***SbK**+**O***	***SbERECTA***
Pn	–								
E	0.99	–							
WUE	0.74	−0.57	–						
VPD	0.88	0.92	−0.68	–					
*SbNADP-ME*	0.96	1.00	−0.90	0.94	–				
*SbCA*	0.79	0.88	−0.73	0.76	0.90	–			
*SbDHN*	0.97	1.00	−0.97	0.95	1.00	0.87	–		
*SbK+O*	0.88	0.87	0.23	0.61	0.83	0.83	0.82	–	
*SbERECTA*	0.96	0.98	−0.64	0.98	0.98	0.80	0.99	0.75	–

*Different colors indicate the positive (green) or the negative (red) Pearson correlation found*.

## Discussion

### The discriminating capacity of the whole-canopy multi-chamber system

The high number of measurements that the whole-canopy multi-chamber system records in 1 day (120 for each one of the 12 whole-canopy chambers, one measurement per chamber every 12 min) enabled the identification of relevant physiological parameters. Confirming results obtained with similar systems (Poni et al., 2014; Rodrigues et al., 2016), our system measured effectively the diurnal gas exchange dynamics of both WW and WS plants. Changes in Pn and E induced by air temperature, VPD and soil water availability (FTSW) were continuously and non-destructively measured by the system and gathered in a rich database that was used to confirm the strategies adopted by IS20351 and IS22330 to cope with drought stress, previously hypothesized by Fracasso et al. (2016a,b).

In fact, stress at 0.45 FTSW (47th DAE) induced a decrease in Pn and E only in WS IS20351 plants but not in WS IS22330 plants (Figures 3F,G). This implies that while for IS20351 the value of 0.45 FTSW makes Pn and E sensitive to VPD, for the tolerant genotype IS22330 this value is not yet limiting. This confirms and reinforces results obtained from a previous experiment in which gas exchange were measured at leaf level on the same genotypes (Fracasso et al., 2016a). According to Fracasso et al. (2016a), the threshold value of FTSW, below which E was limited, was higher in the sensitive genotype than in the tolerant one.

The strategy, classified as “pessimistic” according to Jones (1980), adopted by the tolerant IS22330 genotype was evident under severe stress conditions (0.15 FTSW, Figure 3L–N). While E of WS IS20351 plants still increased during the day following the VPD trend (even though to a lower extent compared to *E*-values recorded at 0.45 and 0.70 FTSW), E of WS IS22330 remained stable with a slight decrease around 11 a.m. (Figure 3M), when VPD started to increase.

Since Pn of WS IS20351 and WS IS22330 plants was similar at 0.15 FTSW, the differences encountered in wcWUE_i_ of WS IS20351 and WS IS22330 were due to E, that was mainly affected by VPD. Whole-canopy WUE_i_ values were different from WUE_i_ calculated at leaf level in Fracasso et al. (2016b). While at leaf level WUE_i_ of WS plants decreased according to decreasing FTSW, at whole-canopy level WUE_i_ of WS plants increased with the decreasing FTSW. Our results confirm previous findings of Tomás et al. (2012) and the inadequacy of single-leaf measurements to represent the spatial and temporal variability occurred at whole-canopy level. In addition, our results clearly highlighted that, when water shortage become severe (0.15 FTSW), wcWUE_i_ of WS plants is higher than wcWUE_i_ of WW plants (Figure 3N), which is in contrast to what Poni et al. (2014) found in grapevine. The increase in wcWUE_i_ encountered in WS plants was observed also in other species (Condon et al., 2002; Gilbert et al., 2011; Liu et al., 2016)

### Drought related proxy genes

The second objective of this study (b) was to evaluate the gene expression dynamics of five drought related genes, identified in previous experiments (Pasini et al., 2014; Fracasso et al., 2016b), to assess which one explains the larger daily variation of Pn, E, and wcWUE_i_ and therefore to identify a proxy gene for whole-canopy WUE_i_ estimation. The differences in gene expression and their correlation with physiological parameters were extremely evident in this experiment (Table 2).

Coding for the NADP-malic enzyme and for a carbonic anhydrase, *SbNADP-ME* and *SbCA* are the most important enzymes in the C_4_ cycle involved respectively in the production of NADPH and in the provision of HCO^3−^ to phosphoenolpyruvate carboxylase (PEPC), the latter involved also in the control of stomata aperture (Hu et al., 2010). Plants transformed with *NADP-ME* showed increased drought avoidance and soil water conservation (Guo et al., 2009; Doubnerová and Ryšlavá, 2011). The correlation existing between high NADP-ME expression level and low stomatal conductance reported by Laporte et al. (2002), was confirmed in the present study. The drought tolerant genotype IS22330 had, in fact, higher NADP-ME expression level than the sensitive IS20351, that resulted in a lower Pn and E compared to IS20351. CA has been proposed, together with aquaporins family, as responsible for rapid regulation of g_m_ (conductance of mesophyll) (Perez-Martin et al., 2014). CA activity are directly proportional to g_m_ even if this relation is species dependent (Gillon and Yakir, 2001). The low gene expression value of *SbCA*, recorded in the drought tolerant IS22330 genotype and reflected in the lower Pn and *E*-values recorded under severe drought stress conditions (0.15 FTSW), makes this gene a potentially useful proxy for drought tolerance screening. The daily trends of *SbNADP-ME* and *SbCA* expression confirmed that the strategy adopted by IS22330 to cope with drought, relies on the accurate control of stomatal openings, which confirms what was previously hypothesized by Fracasso et al. (2016a).

*SbDHN* is involved in the dehydrin biosynthetic pathway and codes for one of the hydrophilic globular proteins called also late embryogenesis abundant (LEA) proteins. These proteins accumulate in response to drought stress protecting plants by maintaining the cell membrane structure and ion balance (Gosal et al., 2009), or acting as molecular chaperones (Chandra Babu et al., 2004). As recently showed (Fracasso et al., 2016a), *SbDHN* could be a suitable tools to screen sorghum genotypes for drought tolerance. This is further confirmed in the present study where, under severe drought stress conditions, *SbDHN* was more expressed in the drought tolerant genotype IS22330 than in the sensitive IS20351, and was more strongly correlated to *SbERECTA* (with positive correlation) and to whole-canopy WUE_i_ (with negative correlation) in the tolerant IS22330 than in the IS20351.

The opening of stomata apertures requires the uptake of potassium ions into guard cells. The responsible of this uptake are potassium channels and pumps that have been identified on the cell membrane of the guard cells (Schroeder et al., 1984). Ion release from guard cells causes, on the other hand, the closure of stomata apertures. Other ion channels are involved in the release of ions from guard cells, causing an osmotic efflux of water out of the guard cells and the following cell shrinkage and closure of stomata apertures (Keller et al., 1989; Thiel et al., 1992). The higher expression level of *SbK*+*O*, coding for an osmotic stress potassium transmembrane transporter, recorded in the drought tolerant IS22330 compared to the sensitive IS20351, and the stronger correlation of this gene with Pn and E in IS22330 than in IS20351, suggests that the drought tolerant IS22330 is more able to manage water losses through stomata apertures than the sensitive IS20351. The down-regulation of *SbK*+*O* at 2 p.m. (Figure 4D), occurred under WS conditions for both genotype at the same time when VPD reached its maximum level, reflects the strong dependence of *SbK*+*O* expression to the evaporative demand of the atmosphere. Sorghum plants under WS conditions reduce *SbK*+*O* expression level when VPD is maximum.

The daily expression level of a putative leucine-rich repeat receptor-like protein kinase family protein, *SbERECTA*, homologous to *PdERECTA* (Xing et al., 2011) and chosen to test its implication in improving WUE also in *Sorghum bicolor*, was negatively correlated with IS22330 and positively correlated with IS203351 WUE_i_ daily trend. After 10 a.m. this gene was up-regulated in response to severe stress more in the drought tolerant IS22330 than in the drought sensitive IS20351, allowing to speculate on its role in determining wcWUE_i_.

## Conclusions

The multi-chamber system proved to be a valuable tool in discriminating continuously and non-destructively the whole-canopy Pn, E, and WUE_i_ of two sorghum genotypes in response to progressive drought stress. The drought tolerant genotype IS22330, with its low Pn and E under WS conditions, adopts a “pessimistic” strategy, limiting water consumption and achieving overall a wcWUE_i_ higher than the drought sensitive IS20351. This drought tolerance strategy is pursued through a fine control of stomata apertures involving regulation of K^+^ channels expression levels (*SbK*+*O*) and regulation of C4 cycle gene expression levels (*SbNADP-ME* and *SbCA*).

The positive correlation between *SbCA* expression level and Pn and E indicates that *SbCA* could be considered a candidate gene for drought tolerance improvement in sorghum. *SbERECTA*, on the other hand, could be used as a proxy for wcWUE_i_ determination also in sorghum while *SbDHN* was confirmed as a valuable candidate to assess drought tolerance in sorghum throughout the day. On the contrary, *SbNADP-ME, SbCA, SbK*+*O*, and *SbERECTA* changed their expression according to VPD. The real-time non-destructive determination of whole-canopy Pn and E, together with wcWUE_i_ estimation, were useful to accurately identify at what time the destructive sampling had to be done in order to identify significantly different gene expression levels between genotypes and significant correlation with physiological measurements. The best time to carry out a destructive sampling for gene expression analysis, in order to assess drought tolerance in sorghum, is when VPD is maximum.

## Author contributions

AF and SA conceived and planned the experiment. EM and AF set up and monitored the canopy gas exchange system. AF performed the experimental work, data analysis and drafted the manuscript. SA and AM revised the manuscript. SA supervised the research. All the authors have read and approved the final manuscript.

### Conflict of interest statement

The authors declare that the research was conducted in the absence of any commercial or financial relationships that could be construed as a potential conflict of interest.
